# Supplementary material for: Community standards for open cell migration data
Source: Gigascience. 2020 May 12;9(5):giaa041. doi: 10.1093/gigascience/giaa041 (PMC7317087; doi:10.1093/gigascience/giaa041)
Supplement: giaa041_GIGA-D-19-00389_Revision_1 [file giaa041_giga-d-19-00389_revision_1.pdf]

GigaScience

Community Standards for Open Cell Migration Data

--Manuscript Draft--

**Manuscript Number:**	GIGA-D-19-00389R1
**Full Title:**	Community Standards for Open Cell Migration Data
**Article Type:**	Review
**Funding Information:**	
**Abstract:**	Cell migration research has become a high-content field. However, the quantitative information encapsulated in these complex and high-dimensional datasets is not fully exploited due to the diversity of experimental protocols and non-standardised output formats. In addition, typically the datasets are not open for reuse. Making the data open and Findable, Accessible, Interoperable, and Reusable (FAIR) will enable meta-analysis, data integration, and data mining. Standardised data formats and controlled vocabularies are essential for building a suitable infrastructure for that purpose but are not available in the cell migration domain. We here present standardisation efforts by the Cell Migration Standardisation Organization, CMSO, an open community-driven organisation to facilitate the development of standards for cell migration data. This work will foster the development of improved algorithms and tools, and enable secondary analysis of public datasets, ultimately unlocking new knowledge of the complex biological process of cell migration.
**Corresponding Author:**	Lennart Martens<br><br>BELGIUM
**Corresponding Author Secondary Information:**	
**Corresponding Author's Institution:**	
**Corresponding Author's Secondary Institution:**	
**First Author:**	Alejandra N. Gonzalez-Beltran
**First Author Secondary Information:**	
**Order of Authors:**	Alejandra N. Gonzalez-Beltran
	Paola Masuzzo
	Christophe Ampe
	Gert-Jan Bakker
	Sebastien Besson
	Robert H. Eibl
	Peter Friedl
	Matthias Gunzer
	Mark Kittisopikul
	Sylvia E. Le Devedec
	Simone Leo
	Josh Moore
	Yael Paran
	Jaime Prilusky
	Philippe Rocca-Serra
	Philippe Roudot
	Marc Schuster
	Gwendolien Sergeant

Powered by Editorial Manager® and ProduXion Manager® from Aries Systems Corporation

	Staffan Stromblad
	Jason R. Swedlow
	Merijn van Erp
	Marleen Van Troys
	Assaf Zaritsky
	Susanna-Assunta Sansone
	Lennart Martens
**Order of Authors Secondary Information:**	
**Response to Reviewers:**	We have responded to each of the reviewer comments in the Response Document added to the submission, and indicated the changes performed to the manuscript. We thank the reviewers again for their comments, which have helped us improve the manuscript.
**Additional Information:**	
**Question**	**Response**
Are you submitting this manuscript to a special series or article collection?	No
**Experimental design and statistics**<br><br>Full details of the experimental design and statistical methods used should be given in the Methods section, as detailed in our Minimum Standards Reporting Checklist. Information essential to interpreting the data presented should be made available in the figure legends.<br><br>Have you included all the information requested in your manuscript?	Yes
**Resources**<br><br>A description of all resources used, including antibodies, cell lines, animals and software tools, with enough information to allow them to be uniquely identified, should be included in the Methods section. Authors are strongly encouraged to cite Research Resource Identifiers (RRIDs) for antibodies, model organisms and tools, where possible.<br><br>Have you included the information requested as detailed in our Minimum Standards Reporting Checklist?	Yes

**Availability of data and materials**<br><br>All datasets and code on which the conclusions of the paper rely must be either included in your submission or deposited in publicly available repositories (where available and ethically appropriate), referencing such data using a unique identifier in the references and in the “Availability of Data and Materials” section of your manuscript.<br><br>Have you have met the above requirement as detailed in our Minimum Standards Reporting Checklist?	Yes

Powered by Editorial Manager® and ProduXion Manager® from Aries Systems Corporation

Revised manuscript with track changes on, without figures
Click here to access/download;Manuscript;Community Standards for Open Cell Migration Data.docx

Community Standards for Open Cell Migration Data

Alejandra N. Gonzalez-Beltran[1,2,§] (ORCID: 0000-0003-3499-8262)

Paola Masuzzo[3,4,5,§] (ORCID: 0000-0003-3699-1195)

Christophe Ampe[4] (ORCID: 0000-0002-8554-3594)

Gert-Jan Bakker[6] (ORCID: 0000-0003-3602-0014)

Sébastien Besson[7] (ORCID: 0000-0001-8783-1429)

Robert H. Eibl[8] (ORCID: 0000-0002-6687-5286)

Peter Friedl[6,9,10] (ORCID: 0000-0002-0119-4041)

Matthias Gunzer[11, 12~~21~~] (ORCID: 0000-0002-5534-6055)

Mark Kittisopikul[1~~2~~3,1~~3~~4] (ORCID: 0000-0002-9558-6248)

Sylvia E. Le Dévédec[1~~4~~5] (ORCID: 0000-0002-0615-9616)

Simone Leo[7,1~~5~~6] (ORCID: 0000-0001-8271-5429)

Josh Moore[7] (ORCID: 0000-0003-4028-811X)

Yael Paran[1~~6~~7] (ORCID: 0000-0001-9544-6213)

Jaime Prilusky[1~~7~~8] (ORCID: 0000-0002-7019-0191)

Philippe Rocca-Serra[1] (ORCID: 0000-0001-9853-5668)

Philippe Roudot[1~~8~~9] (ORCID: 0000-0001-6632-8728)

Marc Schuster[11] (ORCID: 0000-0002-7751-7708)

Gwendolien Sergeant[4] (ORCID: 0000-0001-9869-3826)

Staffan Strömblad[~~19~~20] (ORCID: 0000-0002-1236-6339)

Jason R. Swedlow[7] (ORCID: 0000-0002-2198-1958)

Merijn van Erp[6] (ORCID: 0000-0002-9642-9789)

Marleen Van Troys[4] (ORCID: 0000-0003-4283-8103)

Assaf Zaritsky[2~~0~~1] (ORCID: 0000-0002-1477-5478)

Susanna-Assunta Sansone[1*] (ORCID: 0000-0001-5306-5690)

Lennart Martens[3,4, *] (ORCID: 0000-0003-4277-658X)

[§] contributed equally to this work

[*] corresponding authors

susanna-assunta.sansone@oerc.ox.ac.uk

lennart.martens@vib-ugent.be

Author affiliations

[1] Oxford e-Research Centre, Department of Engineering Science, University of Oxford, Oxford, UK;

[2] *current affiliation*: Scientific Computing Department, Rutherford Appleton Laboratory, Science and Technology Facilities Council, Didcot, UK

[3] VIB-UGent Center for Medical Biotechnology, Ghent, Belgium

[4] Department of Biomolecular Medicine, Ghent University, Ghent, Belgium

[5] Institute for Globally Distributed Open Research and Education (IGDORE), Ghent, Belgium

[6] Department of Cell Biology, Radboud Institute for Molecular Life Sciences, Nijmegen, The Netherlands

[7] Centre for Gene Regulation & Expression & Division of Computational Biology, University of Dundee, Dundee, Scotland, UK

[8] German Cancer Research Center, DKFZ Alumni Association, Heidelberg, Germany

[9] David H. Koch Center for Applied Genitourinary Medicine, UT MD Anderson Cancer Center, Houston, TX, USA

[10] Cancer Genomics Center, Utrecht, The Netherlands

[11] Institute for Experimental Immunology and Imaging, University Hospital, University Duisburg-Essen, Essen, Germany

12 Leibniz Institute for Analytical Sciences, ISAS, Dortmund, Germany

[1~~2~~3] Department of Biophysics, UT Southwestern Medical Center, Dallas, TX, USA

[1~~3~~4] Department of Cell and Developmental Biology, Feinberg School of Medicine, Northwestern University, Chicago, IL, USA

[1~~4~~5] Division of Drug Discovery and Safety, Leiden Academic Centre for Drug Research, Leiden University, the Netherlands

[1~~5~~6] Center for Advanced Studies, Research, and Development in Sardinia (CRS4), Pula(CA), Italy

[1~~6~~7] IDEA Bio-Medical Ltd, Rehovot, Israel

[1~~7~~8] Life Science Core Facilities, Weizmann Institute of Science, Rehovot, Israel

[1~~8~~9] Lyda Hill Department of Bioinformatics, UT Southwestern Medical Center, Dallas, TX, USA

[~~19~~20] Department of Biosciences and Nutrition, Karolinska Institutet, Huddinge, Sweden

[2~~0~~1] Department of Software and Information Systems Engineering, Ben-Gurion University of the Negev, Beer-Sheva, Israel

12[~~21~~] ~~Leibniz Institute for Analytical Sciences, ISAS, Dortmund, Germany~~

Formatted: Superscript

Abstract

Cell migration research has become a high-content field. However, the quantitative information encapsulated in these complex and high-dimensional datasets is not fully exploited due to the diversity of experimental protocols and non-standardised output formats. In addition, typically the datasets are not open for reuse. Making the data open and Findable, Accessible, Interoperable, and Reusable (FAIR) will enable meta-analysis, data integration, and data mining. Standardised data formats and controlled vocabularies are essential for building a suitable infrastructure for that purpose but are not available in the cell migration domain. We here present standardisation efforts by the Cell Migration Standardisation Organization, CMSO, an open community-driven organisation to facilitate the development of standards for cell migration data. This work will foster the development of improved algorithms and tools, and enable secondary analysis of public datasets, ultimately unlocking new knowledge of the complex biological process of cell migration.

Keywords: cell migration, data standards, metadata, CMSO, MIACME, biotracks, frictionless data package, FAIR data

Introduction: Towards FAIR and open cell migration data

Due to advances in molecular biology, microscopy technologies and automated image analysis, cell migration research currently produces spatially and temporally resolved, complex and large

datasets. Consequently, experimental imaging techniques have *de facto* entered the “big data” era[1,2]. This creates, on the one hand, challenges[3] for standardising and maintaining data-driven cell migration research in public repositories while, on the other hand, offers unprecedented opportunities for data integration, data mining, and meta-analyses.

This situation resembles the progress that has been made in the *omics* fields integrating standardised data generation, sharing and analysis over the last two decades[4,5]. The ultimate goal for cell migration data processing is to follow a similar route to progress and become more quantitative, interdisciplinary and collaborative.

To enable cell migration data integration, mining and meta-analysis, we initiated an open data exchange ecosystem for cell migration research[6]. The aim was to overcome the current fragmentation of cell migration research and facilitate data exchange, dissemination, verification, interoperability and reuse, as well as encourage data sharing[7]. This should also increase the reproducibility of experiments, enable data mining and meta-analyses, and thus satisfy the FAIR principles for Findable, Accessible, Interoperable and Reusable data[8]. Public availability of both cell migration data and metadata, and designated tools to mine these data, will facilitate the understanding of complex cell functions and their relevance for clinical use in health and disease. In addition, it will attract computational scientists to the field, producing *in silico* models allowing numerical hypotheses to be tested experimentally[9,10].

Establishing such an open cell migration data ecosystem needs community consensus on what content to report, what terminologies to use and what structured machine-readable formats to use in order to represent the experimental details, workflows and analysis results. A significant challenge is the inherent heterogeneity between experimental data: experiments are performed in a wide array of assays, at all levels of throughput, in diverse cellular models, maintained in

various microenvironments, using multiple microscopy techniques and analysis methods. For example, a common read-out in a cell migration experiment is the measurement of the movement over time of cells and/or subcellular compartments. Other quantitative readouts include cellular morphology and its temporal dynamics[11]. However, there is no standard way to report this information, preventing the integration and mining of these data for downstream knowledge extraction. In addition, usually other experimental details are presented in narrative form in manuscripts, in line with publication policies of scientific journals, and typically not delivered in a uniform machine-readable form. While the 'Methods' section of scientific publications is supposed to enable full understanding of the experimental procedures and support replication, similar experiments may be described in an inconsistent manner in different studies. The methods description may be partial and may leave room for multiple interpretations of the experimental details and procedures of data analysis.

With the ultimate aim of an open data ecosystem, the Cell Migration Standardisation Organization (CMSO) was established in 2016 to define and implement standards for the cell migration community. The CMSO operates openly and transparently, is based on voluntary efforts from the community, and is open to anyone interested in contributing and/or providing feedback. The developed standards are designed and implemented aiming to achieve participants' consensus. The CMSO outputs can be found in GitHub (https://github.com/CellMigStandOrg), while general information and activities are available from the CMSO website (http://cmso.science).

The cell migration community standards are composed of three modules, corresponding to the CMSO working groups (WGs) (**Figure 1**):

1. Reporting guidelines specifying the minimal information required when describing cell migration experiments and data (WG1);
2. Controlled Vocabularies (CVs) that unambiguously annotate these units of information (WG2);
3. Standard file formats for data and metadata, embodying the minimum reporting requirements and CV specifications, and Application Programming Interfaces (APIs), which ensure that all data, results and associated metadata can be read and interpreted by relevant software packages (WG3).

Figure 1 - The Cell Migration Standardisation Organization, CMSO. The three working groups (WGs) deliver specific standards in an interactive manner.

While CMSO consists of these three working groups (WGs) (**Figure 1**), interactions and synergies between them were essential to achieve the integrated model. For example, the information elements identified by the minimum reporting checklist (WG1) were annotated with the terms identified as CVs (in WG2), and both checklist and vocabularies were considered when developing formats and APIs (WG3).

In this article, we introduce the CMSO standards framework, providing an audit trail from the data to the machine-readable and interoperable metadata in a harmonised manner.

Results: CMSO standards and tools

Minimal reporting guidelines and controlled vocabularies

Minimal reporting guidelines, also termed “requirements” or “checklists”, aim to ensure that necessary and sufficient metadata are provided to enable the comprehension of an experiment, future data integration, data mining, and to ensure reproducibility. The CMSO defined iterative versions of the Minimum Information About a Cell Migration Experiment (MIACME) guidelines, the latest version being MIACME 1.1[12] (http://cmso.science/MIACME/, also registered[13] in the FAIRsharing portal[14]). Reporting guidelines, when enforced by journals, are an important factor to boost reproducibility according to 69% of researchers surveyed by Nature[15] and have also been proven to improve the quality of experimental reporting[16].

MIACME consists of: (i) generic information about an investigation, which can involve one or more studies, the associated publications, people, organizations and grants, and (ii) specific information about the associated cell migration experiments. The cell migration-specific part of MIACME is partitioned into three conceptual domains (**Figure 2**): (1) the experimental setup: the assay, cell model, environmental conditions and perturbations; (2) the imaging condition: the microscopy settings; and (3) the data: the raw images, summary information about the data (e.g., number of replicates, number of images), processed images and the derived quantitative analysis outputs.

Figure 2 - Overview of the cell migration-specific part of the MIACME specification (version 1.1[13]).

The figure presents an overview of the three main components of the cell migration experiments

information: experimental setup, imaging condition and data. For more details about the MIACME guidelines, including the interrelationships between the three components, see the associated spreadsheet and schemas, which specify the parameters for each conceptual area together with their requirement level, and illustrate them with examples.

MIACME is presented as a specification accompanied by a spreadsheet that describes entities and properties, their expected values, cardinalities and requirement levels. In addition, we also provide a machine-readable and actionable representation that can be validated in the form of JSON-schemas (https://json-schema.org/) and configurations (or templates) for the ISA-Tabular format, so that MIACME-compliant metadata can be created using the ISA framework[17] (see more details in the next section).

While the minimum information requirements determine the metadata elements to be reported, the community also needs to agree on the terms that will be used when describing cell migration experiments. A controlled vocabulary (CV) provides a standard terminology with unambiguous meaning for a particular domain with the goal of promoting consistent use of terms within a community[18]. These terms can then be included in an ontology that defines formal relationships between them. CVs and ontologies harmonise the data representation to perform queries across data repositories, enable data interoperability and facilitate data integration, data mining and knowledge discovery. A typical data mining study could be based upon a set of competency questions, such as: (i) find all *in vitro* cell migration experiments that make use of live-cell imaging, (ii) retrieve all experiments for which speed was recorded for cells migrating in a 3D collagen matrix, (iii) what is the migratory effect of knockdown of gene X in a breast cancer cell line? (iv) what is the dose response of compound Y on cell line Z in an invasion experiment?

The scope of a CV is therefore defined by a list of use cases as above. For cell migration, a CV requires, for example, terms for the cell line or type, gene names, and specific compounds used for molecular intervention as well as terms describing the type of cell migration assay or the manner in which the cells are presented at the start of the assay (currently termed *cellInput* in the specification). For particularly complex experiments, additional specific terms may be needed. For instance, for single-cell chemotaxis experiments, the CV needs to include terms for the directional chemoattractant application and the type of microscopy used.

The CMSO recommends the use of multiple ontologies for reporting cell migration experiments. The selection of relevant ontologies was based on an iterative strategy, as follows.

1. Determining the domain and scope of the terminologies, through a list of possible queries used for data mining in the domain, such as those mentioned above.
2. Reusing existing terminologies. Besides being more effective, reusing terminologies is also a best practice and a requirement to interoperate with other applications that have already committed to particular CVs. The CMSO has identified existing controlled terminologies, recognized and maintained by the scientific community, which contain terms relevant for cell migration (see https://fairsharing.org/collection/CellMigrationStandardisationOrganisation).
3. Identifying missing terms (related to cell migration), specifying their definitions and relationships with existing terms. These terms are submitted to existing ontologies, when relevant, or will be created if necessary.

Standard Formats, APIs and tools

Once the content and terminology for reporting cell migration experiments have been defined, the community needs to reach consensus on the definition of a data exchange format to enable

data sharing across researchers, institutes, software tools and data repositories, as well as software libraries and APIs to interact with this format.

A single overarching file format may not suffice to capture the full complexity of the cell migration associated data, and therefore CMSO opted for a collection of well-defined, open file formats. Each format is optimized toward different aspects of the experimental pipeline: (i) experimental metadata, (ii) imaging acquisition and (iii) data analysis routines (**Figure 3**). The existing APIs and formats from the Investigation/Study/Assay (ISA)[17,19] and the Open Microscopy Environment (OME)[20] are used, respectively, for the experimental metadata and the image acquisition (left and middle boxes in **Figure 3**).

Figure 3 - The first standardisation products assembled and developed by CMSO WG3. Experimental setup: Investigation Study Assay (**ISA)**; Image data and metadata: Open Microscopy Environment (**OME)** ; Analytical results: **biotracks**.

The ISA model (http://isa-tools.org, http://isa-specs.readthedocs.org) provides a rich description of the experimental metadata (e.g., sample characteristics, technology and measurement types, sample-to-data relationships). This ISA feature served as basis for the conceptual 'experimental setup' section in the MIACME guidelines (**Figure 2**, pink/left box).

The OME Data Model (http://www.openmicroscopy.org/) is a specification for the exchange of image data. It represents images as 5D entities: the 2D plane (x,y), the focal position (z), the spectral channel, and the time. The OME format also includes metadata such as details of the acquisition system and experimental parameters related to acquisition. These metadata are related to the 'imaging condition' conceptual area within MIACME (**Figure 2**, middle box).

With the ISA and OME models established and publicly available, the remaining challenges are around standardised reporting of routine analyses, such as cell tracking or quantification of cell shape. Here, we report the specification and implementation of a new open tracking data format named **biotracks** (http://cmso.science/Tracks/**, Figures 3 and 4**). The **biotracks** format was designed to accommodate the time-resolved tracking information of various objects observed in cell migration experiments. These tracked objects can either be cells, specific organelles or cellular structures (e.g., leading cell edges, nuclei, microtubule organizing centers, filaments, single molecules, and signals that report signaling dynamics). Generally, an object could be any region of interest (ROI), including an arbitrary mask or even a single point in space. As such, the specification includes three levels of information: (i) objects identified during cell segmentation or detection tasks, (ii) links that linearly connect objects across frames of the acquired time sequence, and (iii) tracks that connect links across events such as splitting or merging (**Figure 4A**). This abstraction enables the standardised description of a wide variety of biological tracking data.

Figure 4 - Schematic view of the biotracks format developed by the CMSO. In **A**, a segmentation algorithm identifies objects in the raw images, annotating them with the frame information, coordinates and any other features the algorithm extracts. These are described in the objects table in **B**. A linking algorithm then connects the objects across frames in a parent-child relationship. Among the possible events, the linking algorithm can then identify a split, where a parent has more than one child. This information is reported in the links table in **B**. The tracks table in **B** can finally be inferred from the objects

and links tables. The tabular data package format is represented in **C**. Here, objects, links and tracks data tables are saved as comma-separated-values (CSV) files. The accompanying file in the JSON format contains both the general metadata of the data package and the metadata of the CSV files.

Whereas **biotracks** follows the general strategy of any tracking software, its specification focuses on enabling data interoperability in a simple way by specialising the Tabular Data Package (http://specs.frictionlessdata.io/tabular-data-package) container format. In the data package, the data (objects, links, and optionally tracks, **Figure 4B**) are stored in tabular form as comma-separated-values (CSV) files, while metadata and schema information are stored as a JavaScript Object Notation (JSON) file (**Figure 4C**). The development of the **biotracks** format is complementary to the OMEGA system for particle tracking data, which has particular emphasis on results from viral and vesicular trafficking experiments[21].

Standardisation is also required in other parts of the experimental process, such as standards to report how the analytical results were obtained; methods for segmentation and other image processing tasks; descriptions of post-image data exclusion and curation and descriptions of statistical analyses[22]. These aspects are left for future work by the CMSO community.

Each of the above mentioned formats (ISA, OME, and biotracks) have associated software to manipulate them, which we introduce below, together with other APIs and tools that facilitate in building machine-actionable and FAIR cell migration data.

The ISA model has an associated set of open source tools (http://isa-tools.org). In particular, the ISAcreator desktop-based tool allows for the creation, parsing and validation of experiments described with the ISA model. A version of ISAcreator is made available including MIACME configurations or templates (https://github.com/CellMigStandOrg/ISAcreator-MIACME). On the

other hand, the ISA-API Python-based software (https://github.com/isa-tools/isa-api) supports the programmatic creation and manipulation of experimental metadata.

The OME model for imaging metadata is supported by several software packages, most notably the Java Bio-Formats library[23], which can read and write OME's own OME-TIFF standard as well as convert a wide variety of proprietary file formats into OME-TIFF (https://docs.openmicroscopy.org/bio-formats/5.9.2/supported-formats.html) and, more recently, OME-Files[24], which serves as a reference implementation of OME-TIFF in C++ and Python.

For the analytical routines downstream, we have developed a library for cell tracking data: the **biotracks** API[25] (https://github.com/CellMigStandOrg/biotracks). As shown in **Figure 5**, the library takes as input a cell tracking file from a tracking software (such as TrackMate[26], CellProfiler[27], Icy[28] and MosaicSuite[29]) and produces a data package where objects and links are stored in the standardised format depicted in **Figure 4**.

Figure 5 - The biotracks library. The library receives cell tracking data as input from multiple tracking software and converts them to the **biotracks** format (see **Figure 4**), which can be further visualized and analyzed with downstream applications within this framework.

The CellMissy software package[30,31] (https://github.com/compomics/cellmissy), a cross-platform data management and analysis system for cell migration/invasion data, was extended to import and export datasets whose experimental metadata is available in MIACME-compliant ISA-Tab format and whose cell tracking data are represented with **biotracks**.

A cell migration data repository (https://repo.cellmigration.org/) was created, which accepts submissions of experimental metadata in ISA-Tab format compliant with the MIACME guidelines together with raw data submission, and supports searching across the deposited data.

The CMSO standards were incorporated into the WiSoft platform, a commercial software developed and distributed by IDEA Bio-Medical LTD, which consists of two software tools – Athena and Minerva - designed to support the addition of experimental parameters, imaging properties, and analysis modules. This extensibility feature facilitated the adoption of the MIACME elements and allows easy adaptation of algorithms contributed by researchers as part of the ongoing effort of analyzing dynamic biological data.

CMSO Standards in Action

To demonstrate the application of the CMSO standards we applied them to the study by Masuzzo *et al.*[30], which proposed an end-to-end software solution for the visualization and analysis of high-throughput single-cell migration experiments. The authors used two datasets to demonstrate their software. Here we reuse their Ba/F3 cells experiment to demonstrate CMSO standards in action as follows. As a first step, we annotated the data using the MIACME guidelines (v1.1) (see example in **Table 1** in the Supplementary Information). The two columns of this table represent the two layers of information of **Figure 2**: specific entities (column 1) are annotated using (controlled) terms (column 2). As shown through this example, the MIACME schema converts a large imaging-based study into an easy-to-interpret structured description. The resulting metadata are available in Github (https://github.com/CellMigStandOrg/CMSO-datasets/tree/master/cmsodataset0001-masuzzo).

As shown above, when reporting an experiment, researchers will need to complete the information indicated in MIACME, and for those fields that require a controlled vocabulary or ontology term, they will need to select the most appropriate terms, considering the suggested ontologies for each field (e.g., for organisms, MIACME recommends the NCBI Taxonomy). The criterion for selecting a term is to consider the most specific description available in the ontology. If no term exists in current ontologies (shown as [*] in **Table 1** in the Supplementary Information), the CMSO community has been submitting it to a relevant ontology. If there is no relevant ontology, the CMSO community has been gathering the terms to create a specific ontology in the future, if necessary.

This MIACME information is included in an ISA-Tab representation of the dataset, which also expands it with more information about the processes performed in the experiment and their intermediate inputs and outputs.

Secondly, to demonstrate the usage of the data formats and APIs, we have prepared an interactive Jupyter notebook, available at https://github.com/CellMigStandOrg/CMSO-training/blob/master/notebooks/CMSO_PM.ipynb. The notebook uses the set of three software libraries discussed above: ISA-tools to manipulate the experimental setup information, OME-files for the imaging data and metadata, and **biotracks** for the cell tracking data. The pipeline presented in the notebook bundles the three libraries together, showing the interaction of the CMSO standards in a complete experimental and analytical workflow.

Discussion: The overall vision

Genomics, proteomics and structural biology have greatly benefited from well-developed data standards[32] that contributed to rapid progress in these fields. However, in the field of cell migration, the lack of unifying standards and repositories have limited the opportunities to make similar progress. Thus, expensive and difficult-to-generate imaging data are stored at local labs with no further access for the community and with no standardised descriptions of the experiments that generated them. CMSO has taken initiatives to increase accessibility and reproducibility of cell migration data across models, by developing an open access reporting structure that aims to accommodate diverse types and complexities of cell migration data. The use of standardised and unambiguous terminology and structured metadata in reporting experiments will allow other researchers to more easily reproduce these experiments.We consider this effort as a first step towards standardisation of cell migration data, which will facilitate integration, validation and meta-analysis of cell migration data across models, and foster progress across study and model comparison, enabling the validation of new discoveries.

In this work, we presented a framework around community-driven standards and tools, developed by CMSO through an open process, for managing cell migration data along its data life cycle. The CMSO framework relies on established standards for experimental metadata (ISA) and imaging data (OME), both complemented with models and tools developed by CMSO. We introduced reporting guidelines that identify what elements should be reported for cell migration experiments (MIACME), and a format for cell tracking data. We also provide APIs and software tools supporting the description and publication of cell migration experiments, their workflows and results.

Open cell migration data following data standards and the tools to manipulate them will enhance the performance and relevance of the field and deepen insight into this complex biological progress beyond the impact of primary research. This requires future actions and tools moving forward: the community must improve the user-friendliness of the routine processes of data curation, deposition and exchange. It also needs ensuring that the data standards continuously evolve to meet community needs. Eventually, the community must strive toward making maximal use of the data: contributing public data; developing new data-driven computational tools; mining for patterns that can drive new biological hypotheses; testing new hypotheses in experimental, computational, and clinical models; and unlocking new knowledge that drives scientific progress and yields new therapies and strategies to improve health.

Outlook: CMSO sustainability

Proper standards and broad community support are crucial for the establishment of a long term open data sharing ecosystem for cell migration research. Therefore, the output of CMSO is a crucial cornerstone for the implementation of such an ecosystem. The European Union Horizon 2020 MULTIMOT project (https://multimot.org/) has been the initial driving force for the development of CMSO, from establishing the organization to arranging the first meetings, enabling the involvement of the broader cell migration community. From the start CMSO was planned as an independent entity, including people beyond MULTIMOT, with its own governance structure (https://cmso.science/roles-and-responsibilities/) and a community-driven decisions mechanism. MULTIMOT members are required to implement the CMSO standards, and therefore they constitute the first users and quality assurance testers. CMSO members are responsible for the dissemination of the materials produced, and for the sustainability of the organization in the future through funding.

One of the prime goals of CMSO is to raise community awareness on best practices for data stewardship, to promote and disseminate the use of community standards. CMSO is managed and run by volunteers from the community, and is open to participation from anyone interested. Current CMSO participants include cell biologists, immunologists, cancer researchers, medical professionals from laboratory medicine, microscopists, computational biologists, and data scientists (https://cmso.science/how-to-get-involved/).

The CMSO also welcomes cell migration data contributions from the scientific community and provides guidelines for creating MIACME-compliant descriptions of experiments and using CVs to annotate them.

Methods

Compilation of use cases

In order to provide incentives for cell migration researchers to invest the time and effort required to structure their data and make it FAIR, CMSO identified a series of use cases where applying the CMSO framework would enable data integration and data reuse and would drive further scientific discoveries.

Combining harmonised data (e.g. from the same cell culture model retrieved from different studies[33–36]) will facilitate data analysis and mining across imaging acquisition techniques, set-ups and cell lines, with applications such as: (i) comparison of results from 2D and 3D culture environments that make use of the same cell model; (ii) validation of *in vitro* results from against datasets from *in vivo* experiments in model organisms (e.g. zebrafish embryos, mouse models of disease) to ascertain *in vivo* relevance; or (iii) systematic comparison of the relative dose-effects of growth factors or chemical compounds on migration behaviors across cell models.

As an important further opportunity, the reuse of existing primary image data with new analyses can reveal previously unexplored patterns contained in such complex data[33–36]. Typical examples for secondary reuse[7] of multiparametric imaging datasets results from applying novel computational algorithms to derive kinetic shape features (e.g. leading edge oscillations or membrane curvature changes) or functional components such as switch behaviours and stochasticity in cell population behaviour[37,9]. Furthermore, public datasets can serve as benchmarks for comparing the performance of computational and analytical methods. When they include proper metadata annotation, such datasets are invaluable for developing new methods[38–40] and training machine learning algorithms in a variety of analytical tasks [41]. As resources for the development and comparison of methods, the value of large amounts of publicly available image data cannot be overstated[42,43],[44].

FAIR standards for cell migration

Since the turn of the century, there have been standardisation efforts in different domains: genomics[45,46], proteomics[47–49], and metabolomics[50–52]. Traditionally, these efforts considered each of the aspects (content, terminology, formats) independently, see the FAIRsharing repository of standards[32]. Different communities developed minimum information guidelines in narrative form (e.g., MIAME for microarray[53]; MIAPE for proteomics[47]) and while they encouraged the use of ontologies[54] and formats[48] for annotation and representation, respectively, they developed them separately and emphasised that the guidelines were implementation-independent[47]. Whereas independent reporting guidelines imply that they can be implemented in different formats with different semantic models, the importance of producing data that is truly FAIR in cell migration and the breadth of assays and technologies that are used in the field mandates that a reporting guideline in narrative form is no longer sufficient. The need for FAIR data requires a reference implementation, i.e. a standard software tool(s) that

reads and writes in a standardised format that meets the specification, along with clear, usable examples that show how to implement and use the format. Finally, it is essential to provide validation tool(s) so that scientists and technology developers attempting to adopt the standard can be assured their work is compliant with the reporting guidelines. A comprehensive approach that encompasses the content, terminology and format and thus considers the full machine-actionable model for data description is needed. This is the strategy chosen by CMSO. In this way, the minimal reporting checklist is a first step towards the identification of the metadata elements required for a full data description model, especially in view of the FAIR data principles[8] and FAIR data models[55,56]. We chose a variety of formats to represent the checklist, including a machine-actionable and FAIR representation based on JSON-schemas for JSON-LD data.

To produce these models and tools following a community-driven approach, during face-to-face workshops as well as via online tools, we run the following activities:

- Identified metadata descriptors for cell migration experiments based on the model used by the CellMissy software tool. These metadata descriptors were divided into three categories: experimental setup, imaging condition and cell migration data. Then, a group of cell migration researchers ranked the descriptors according to three values representing: important, somewhat important and not so important. The analysis of the ranking provided the first guidance to build the initial MIACME guidelines.
- After choosing a representative paper on cell migration[57] , a survey was prepared and distributed to researchers asking them to complete the values of the identified metadata descriptors considering the experimental description given in the paper. By asking them to complete the values, we could check if it was easy to identify the relevant element in the paper, and how clear the explanation about the descriptor was. The descriptors were split in multiple sections: general experiment overview and description, cell system

description, cell culture conditions, assay description, vessel, plate and environment information, perturbation and intervention, imaging, image analysis information, licensing and terms of use, and request for feedback. Again, we requested researchers to rate each element on a 1 to 5 scale, from essential to useless.

- The above steps and discussions with researchers allowed us to refine the metadata descriptors to be included in MIACME, and in this way we developed several versions of the checklist.
- During this iterative process, we also identified the values for the different elements for a variety of experiments, some published and some unpublished. The feedback and discussions among researchers about the importance of each descriptor was crucial to keep refining the checklist and settle on a set of descriptors deemed minimal but also sufficient to enable the comprehension and replicability of the experiment.
- In terms of semantic annotations, we found terms in existing ontologies when available and otherwise requested the addition of terms in relevant ontologies (e.g. https://github.com/information-artifact-ontology/IAO/issues/212)
- The development of a common standard format to represent cell tracking data associated with cell migration experiments aimed to produce a simple and extensible format, reuse existing standard formats where possible and support both human- and machine-readable metadata. The adopted solution relies on the Tabular Data Package, which supports the association of data with a JSON file to specify metadata and schema information, and resulted in a specification (https://cmso.science/Tracks/) and a python-based software tool (biotracks) to manage the new format.
- We also compiled experimental datasets to demonstrate the application of the different standards and how they integrate in the CMSO framework.

Acknowledgments

The Cell Migration Standardisation Organisation acknowledges funding from the European Union's Horizon 2020 Programme under the MultiMot project, Grant Agreement 634107 (PHC32-2014).

Author Contributions

AGB and PM contributed equally to writing the manuscript and addressed all authors comments and contributions. AGB led the development of the MIACME reporting guidelines, coordinating the community feedback and updates, with contributions from PRS, PM, MVT, AZ, RHE, LM and feedback from cell migration researchers involved in CMSO via face-to-face meetings and online communications. AGB wrote the MIACME specification, PRS and AGB produced MIACME-compliant ISA-Tab configurations. AGB produced MIACME JSON-schemas and JSON-LD context files. PM and SB led the development of the biotracks specification with contributions from SL, GS, JM and the CMSO community. PM led the development of the biotracks package with contributions from SL and GS. GS led the extension of CellMissy to support the CMSO standards. JP developed the cell migration repository. YP extended the IDEA Bio-Medical LTD software to support the CMSO standards. PRS produced the ISA-TAB representation of cell migration studies published by PM. PRS provided the list of recommended ontologies. PM, AGB, SL produced the examples of CMSO datasets with their MIACME, ISA-Tab, OME and biotracks representations and code to validate them. SB, AGB, SL, PM, MVT produced the training material. AGB created the FAIRsharing collection for CMSO and included its widget in the CMSO website. AGB, SB, LM and PM produced the CMSO website. All authors contributed to, read and approved the final manuscript.

Abbreviations

API	Application Programming Interface

CBO	Cell Behaviour Ontology
chEBI	Chemical Entities of Biological Interest
CLO	Cell Line Ontology
CMPO	Cellular Microscopy Phenotype Ontology
CMSO	Cell Migration Standardisation Organization
CSV	Comma Separated Values
CV	Controlled Vocabulary
EFO	Experimental Factor Ontology
ERO	eagle-i Research Resource Ontology
FAIR	Findable, Accessible, Interoperable and Reusable
GO	Gene Ontology
HPO	Human Phenotype Ontology
ISA	Investigation, Study, Assay
JSON	JavaScript Object Notation
JSON-LD	JavaScript Object Notation for Linked Data
JSON-schema	JavaScript Object Notation Schema
MIACME	Minimum Information for Cell Migration Experiments
MIAME	Minimum Information About a Microarray Experiment
MIAPE	Minimum Information About a Proteomics Experiment

NCBITaxon	NCBI Taxonomy
OBI	Ontology for Biomedical Investigation
OME	Open Microscopy Environment
OME-TIFF	Open Microscopy Environment Tagged Image File Format
ROI	Region of Interest
UO	Units Ontology
WG	Working Group

Supplementary Information

Table 1: The cell migration study from Masuzzo *et al*.[30] described and annotated using the cell-migration-specific part of the MIACME guidelines (version 1.1). Highlighted in gray, we show elements that are part of the MIACME requirements but were not reported in the original paper.

**Experimental setup**	
**Entity**	**Term [CV]**
Basic Approach	*In vitro* design [OBI:0001285]
Cell Model	Cell type:<br>cell line cell [CLO:0000001]
	Specific Cell Details:<br>B-cell [CL:0000236]<br>Ba/F3 cell [CLO:0001842]

	Organism: Mus musculus [NCBITaxon:10090]
Assay	Assay type: Single-cell migration [*]<br>Cell Input: single cells [*]
Treatment	Factor: Bcr-Abl fusion protein [PR:000044437] oncogene variants[58]
	Factor: ROCK inhibitor y-27632 [CHEBI:75393]<br>Factor level: 10 µM
Microenvironment	BD Matrigel™ Basement Membrane [GO:0005604,NCIT_C19834] Matrix
Medium	RPMI 1640 + FBS [fetal bovine serum] [CCONT:0000048][+]
**Imaging condition**	
**Entity**	**Term [CV]**
Imaging Modality	Phase-contrast [ERO:0001636]
Image Sequence Type	Time series / Time-lapse [FBbi_00000249]
Observation Period	6
Observation Period Unit	hours
Time Series Interval	1.5
Time Series Interval Unit	minutes
Objective Lens	Manufacturer: Olympus<br>Acronym: LCPLFLN<br>Magnification: 20x
Numerical Aperture	0.45

Pixel	Pixel dimension order: XYTZC<br>Pixel type: int16<br>Pixel size X: 1376<br>Pixel size Y: 1038<br>Pixel size T: 240<br>Pixel size C: 1<br>physicalSizeX: 0.645<br>physicalSizeXUnit: µm<br>physicalSizeY:0.645<br>physicalSizeYUnit: µm
Channel Definitions	Channel #1: Cells [CL:0000000]
**Data**	
**Entity**	**Term [CV]**
rawImagesDataset<br>description<br>numberOfFiles	144 raw images x 12 wells<br>1728
processedImagesDataset description<br>keyword	Segmented images/images with tracks [NCIT_C80146]
extractedFeaturesDataset description<br>keyword<br>keyword<br>keyword	Extracted features in the study.<br>X coordinate [NCIT_C44477]<br>Y coordinate [NCIT_C44478]<br>Timepoint [NCIT_C68568]

[*] a CV is not present in literature to describe the term and will be addressed by CMSO.

[+] the cell culture ontology has the term we need, but it has been down for some time; the developer indicated that due to GDPR issues, they are revising the webpages and might be down for a while. If not restored, this term would be a candidate for creation within a CMSO ontology.

Bibliography

1. Meijering, E., Carpenter, A. E., Peng, H., Hamprecht, F. A. & Olivo-Marin, J.-C. Imagining the future of bioimage analysis. *Nat. Biotechnol.* **34,** 1250–1255 (2016).
2. Peng, H. *et al.* Bioimage informatics for big data. *Adv. Anat. Embryol. Cell Biol.* **219,** 263–272 (2016).
3. Macklin, P. Key challenges facing data-driven multicellular systems biology. *Arxiv*
4. Chervitz, S. A. *et al.* Data standards for Omics data: the basis of data sharing and reuse. *Methods Mol. Biol.* **719,** 31–69 (2011).
5. Gomez-Cabrero, D. *et al.* Data integration in the era of omics: current and future challenges. *BMC Syst. Biol.* **8 Suppl 2,** I1 (2014).
6. Masuzzo, P. *et al.* An open data ecosystem for cell migration research. *Trends Cell Biol.* **25,** 55–58 (2015).
7. Zaritsky, A. Sharing and reusing cell image data. *Mol. Biol. Cell* **29,** 1274–1280 (2018).
8. Wilkinson, M. D. *et al.* The FAIR Guiding Principles for scientific data management and stewardship. *Sci. Data* **3,** 160018 (2016).
9. Te Boekhorst, V., Preziosi, L. & Friedl, P. Plasticity of cell migration in vivo and in silico. *Annu. Rev. Cell Dev. Biol.* **32,** 491–526 (2016).
10. Zaritsky, A. Cell biologists should specialize, not hybridize. *Nature* **535,** 325 (2016).
11. Shafqat-Abbasi, H. *et al.* An analysis toolbox to explore mesenchymal migration

heterogeneity reveals adaptive switching between distinct modes. *elife* **5,** e11384 (2016).

12. Gonzalez-Beltran, A. & Rocca-Serra, P. CellMigStandOrg/MIACME: MIACME v1.1. *Zenodo* (2019). doi:10.5281/zenodo.3457561
13. Cell Migration Standardisation Organisation. MIACME; Minimum Information About Cell Migration Experiment. *FAIRsharing.org* (2018). at <http://doi.org/10.25504/FAIRsharing.vh2ye1>
14. Sansone, S.-A. *et al.* FAIRsharing as a community approach to standards, repositories and policies. *Nat. Biotechnol.* **37,** 358–367 (2019).
15. Baker, M. 1,500 scientists lift the lid on reproducibility. *Nature* **533,** 452–454 (2016).
16. Turner, L., Shamseer, L., Altman, D. G., Schulz, K. F. & Moher, D. Does use of the CONSORT Statement impact the completeness of reporting of randomised controlled trials published in medical journals? A Cochrane review. *Syst. Rev.* **1,** 60 (2012).
17. Sansone, S.-A. *et al.* Toward interoperable bioscience data. *Nat. Genet.* **44,** 121–126 (2012).
18. Orchard, S., Montecchi-Palazzi, L., Hermjakob, H. & Apweiler, R. The use of common ontologies and controlled vocabularies to enable data exchange and deposition for complex proteomic experiments. *Pac. Symp. Biocomput.* 186–196 (2005).
19. Rocca-Serra, P. *et al.* ISA software suite: supporting standards-compliant experimental annotation and enabling curation at the community level. *Bioinformatics* **26,** 2354–2356 (2010).
20. Goldberg, I. G. *et al.* The Open Microscopy Environment (OME) Data Model and XML file: open tools for informatics and quantitative analysis in biological imaging. *Genome Biol.* **6,** R47 (2005).
21. Rigano, A. *et al.* OMEGA: a software tool for the management, analysis, and dissemination of intracellular trafficking data that incorporates motion type classification and quality control. *BioRxiv* (2018). doi:10.1101/251850

Commented [1]: These are new references

22. Strömblad, S. & Lock, J. G. Using Systems Microscopy to Understand the Emergence of Cell Migration from Cell Organization. *Methods Mol. Biol.* **1749,** 119–134 (2018).
23. Linkert, M. *et al.* Metadata matters: access to image data in the real world. *J. Cell Biol.* **189,** 777–782 (2010).
24. Leigh, R. *et al.* OME Files - An open source reference library for the OME-XML metadata model and the OME-TIFF file format. *BioRxiv* (2016). doi:10.1101/088740
25. Leo, S. *et al.* CellMigStandOrg/biotracks: Release of biotracks 0.5.0. *Zenodo* (2019). doi:10.5281/zenodo.3355530
26. Tinevez, J.-Y. *et al.* TrackMate: An open and extensible platform for single-particle tracking. *Methods* **115,** 80–90 (2017).
27. Bray, M.-A. & Carpenter, A. E. CellProfiler Tracer: exploring and validating high-throughput, time-lapse microscopy image data. *BMC Bioinformatics* **16,** 368 (2015).
28. Chenouard, N., Bloch, I. & Olivo-Marin, J.-C. Multiple hypothesis tracking for cluttered biological image sequences. *IEEE Trans. Pattern Anal. Mach. Intell.* (2013). doi:377077B0-C943-4376-B928-2872987BC8F1
29. Sbalzarini, I. F. & Koumoutsakos, P. Feature point tracking and trajectory analysis for video imaging in cell biology. *J. Struct. Biol.* **151,** 182–195 (2005).
30. Masuzzo, P. *et al.* An end-to-end software solution for the analysis of high-throughput single-cell migration data. *Sci. Rep.* **7,** 42383 (2017).
31. Sergeant, G., Hulstaert, N., Masuzzo, P. & Gonzalez-Beltran, A. *compomics CellMissy*. (Zenodo, 2019).
32. McQuilton, P. *et al.* BioSharing: curated and crowd-sourced metadata standards, databases and data policies in the life sciences. *Database (Oxford)* **2016,** (2016).
33. Pasquetto, I. V., Randles, B. M. & Borgman, C. L. On the reuse of scientific data. *Data Sci. J.* **16,** (2017).
34. Zaritsky, A. *et al.* Seeds of locally aligned motion and stress coordinate a collective cell

migration. *Biophys. J.* **109,** 2492–2500 (2015).

35. Maiuri, P. *et al.* Actin flows mediate a universal coupling between cell speed and cell persistence. *Cell* **161,** 374–386 (2015).

36. Lavi, I., Piel, M., Lennon-Duménil, A.-M., Voituriez, R. & Gov, N. S. Deterministic patterns in cell motility. *Nat. Phys.* **12,** 1146–1152 (2016).

37. Haeger, A., Krause, M., Wolf, K. & Friedl, P. Cell jamming: collective invasion of mesenchymal tumor cells imposed by tissue confinement. *Biochim. Biophys. Acta* **1840,** 2386–2395 (2014).

38. Zaritsky, A. *et al.* Decoupling global biases and local interactions between cell biological variables. *elife* **6,** (2017).

39. Eibl, R. H. & Benoit, M. Molecular resolution of cell adhesion forces. *IEE Proc. Nanobiotechnol.* **151,** 128–132 (2004).

40. Eibl, R. H. & Moy, V. T. Atomic force microscopy measurements of protein-ligand interactions on living cells. *Methods Mol. Biol.* **305,** 439–450 (2005).

41. Sullivan, D. P. & Lundberg, E. Seeing more: A future of augmented microscopy. *Cell* **173,** 546–548 (2018).

42. Williams, E. *et al.* The image data resource: A bioimage data integration and publication platform. *Nat. Methods* **14,** 775–781 (2017).

43. Sharing images. *Nat. Methods* **14,** 753–753 (2017).

44. Ellenberg, J. *et al.* A call for public archives for biological image data. *Nat. Methods* **15,** 849–854 (2018).

45. Field, D. *et al.* Genomic standards consortium projects. *Stand. Genomic Sci.* **9,** 599–601 (2014).

46. Field, D. *et al.* The minimum information about a genome sequence (MIGS) specification. *Nat. Biotechnol.* **26,** 541–547 (2008).

47. Taylor, C. F. *et al.* The minimum information about a proteomics experiment (MIAPE). *Nat.*

Biotechnol. **25,** 887–893 (2007).

48. Martens, L. *et al.* mzML--a community standard for mass spectrometry data. *Mol. Cell. Proteomics* **10,** R110.000133 (2011).
49. Deutsch, E. W. *et al.* Development of data representation standards by the human proteome organization proteomics standards initiative. *J. Am. Med. Inform. Assoc.* **22,** 495–506 (2015).
50. MSI Board Members *et al.* The metabolomics standards initiative. *Nat. Biotechnol.* **25,** 846–848 (2007).
51. Salek, R. M., Steinbeck, C., Viant, M. R., Goodacre, R. & Dunn, W. B. The role of reporting standards for metabolite annotation and identification in metabolomic studies. *Gigascience* **2,** 13 (2013).
52. Sumner, L. W. *et al.* Proposed minimum reporting standards for chemical analysis Chemical Analysis Working Group (CAWG) Metabolomics Standards Initiative (MSI). *Metabolomics* **3,** 211–221 (2007).
53. Brazma, A. *et al.* Minimum information about a microarray experiment (MIAME)-toward standards for microarray data. *Nat. Genet.* **29,** 365–371 (2001).
54. Mayer, G. *et al.* The HUPO proteomics standards initiative- mass spectrometry controlled vocabulary. *Database (Oxford)* **2013,** bat009 (2013).
55. Data models to GO-FAIR. *Nat. Genet.* **49,** 971 (2017).
56. González-Beltrán, A., Maguire, E., Sansone, S.-A. & Rocca-Serra, P. linkedISA: semantic representation of ISA-Tab experimental metadata. *BMC Bioinformatics* **15 Suppl 14,** S4 (2014).
57. Lock, J. G. *et al.* Plasticity in the macromolecular-scale causal networks of cell migration. *PLoS ONE* **9,** e90593 (2014).
58. Rochelle, T. *et al.* p210bcr-abl induces amoeboid motility by recruiting ADF/destrin through RhoA/ROCK1. *FASEB J.* **27,** 123–134 (2013).

Figure 1

Click here to access/download;Figure;Figure_01_CMSO.png

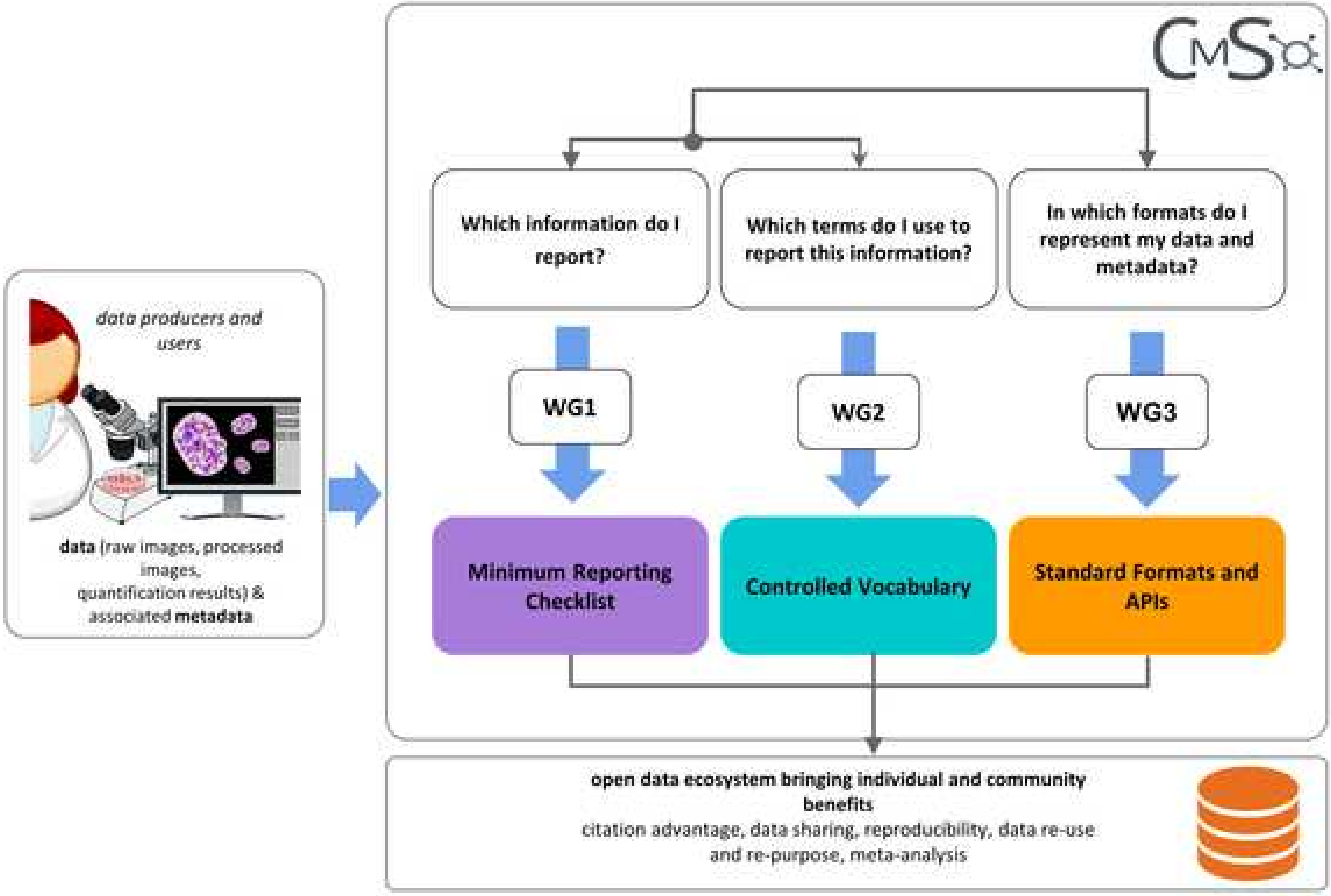

Figure 2

Click here to access/download;Figure;Figure_02_MIACME_1.1.png

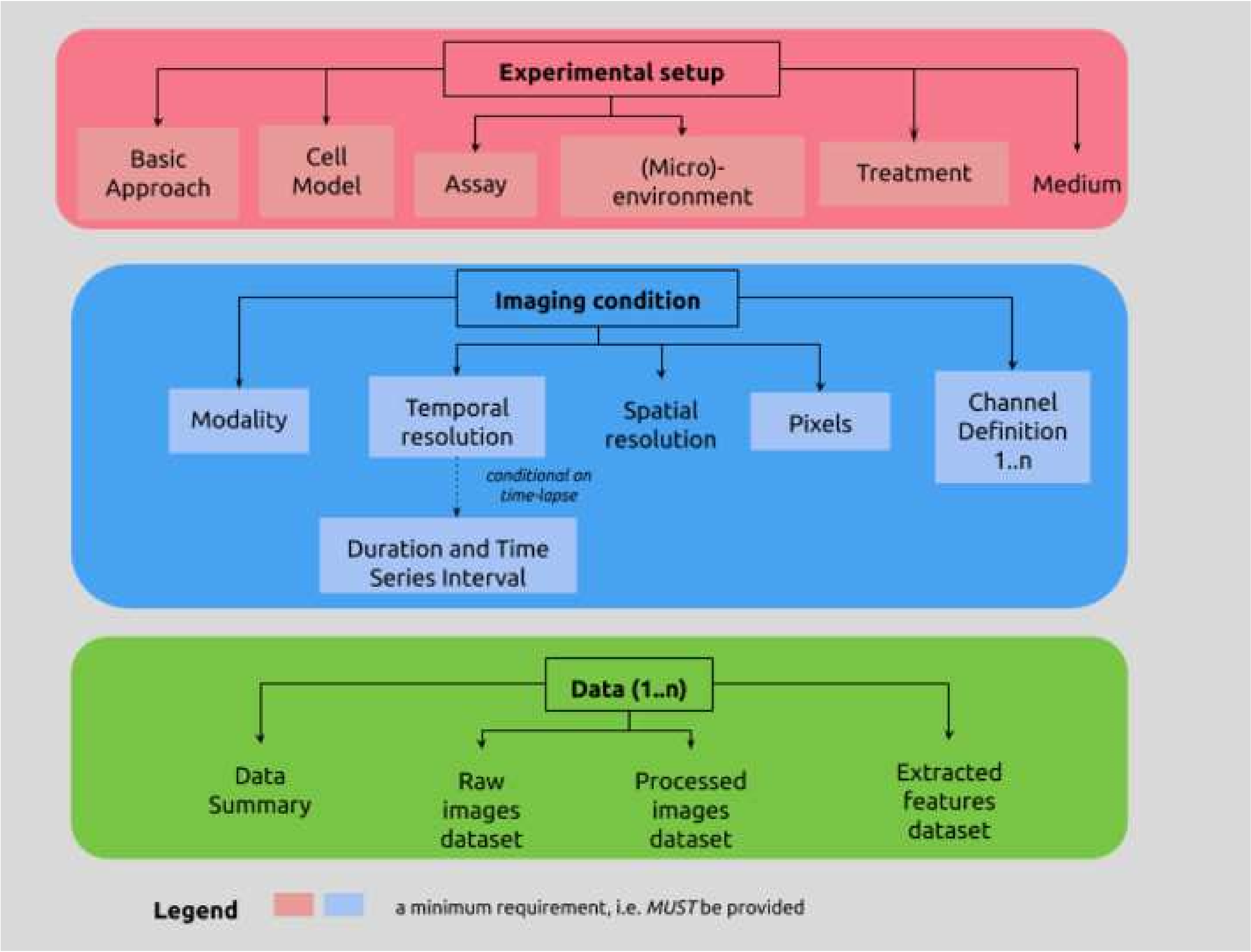

Figure 3

Click here to access/download;Figure;Figure_03_blocks_wg3.png

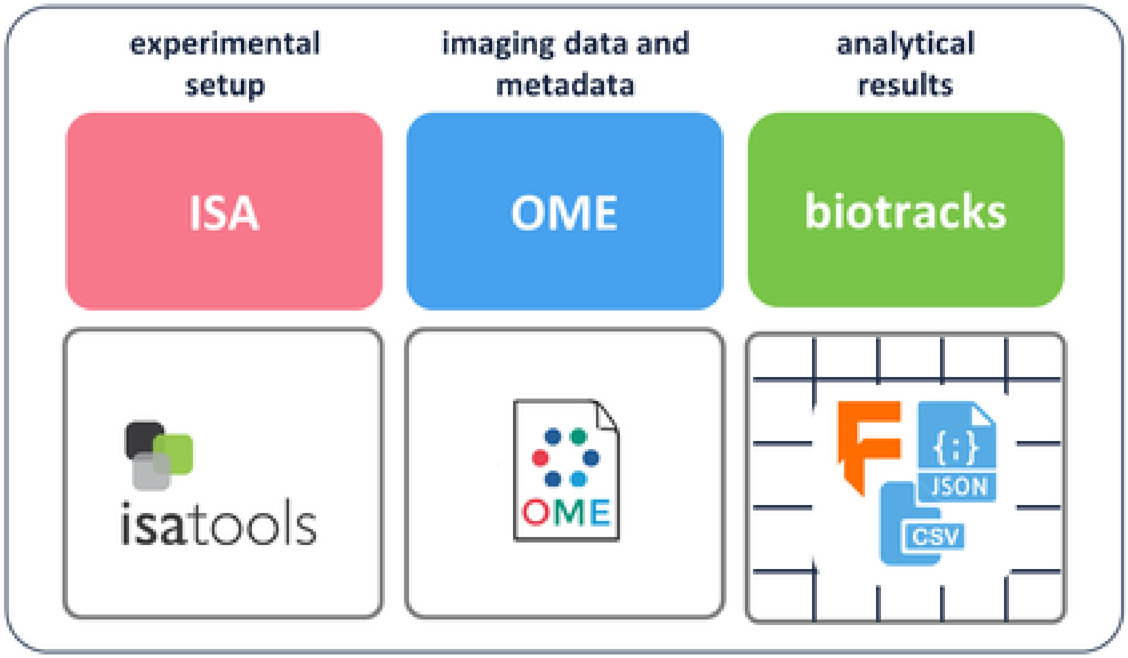

Figure 4

Click here to access/download;Figure;Figure_04_biotracks_schema_full.png

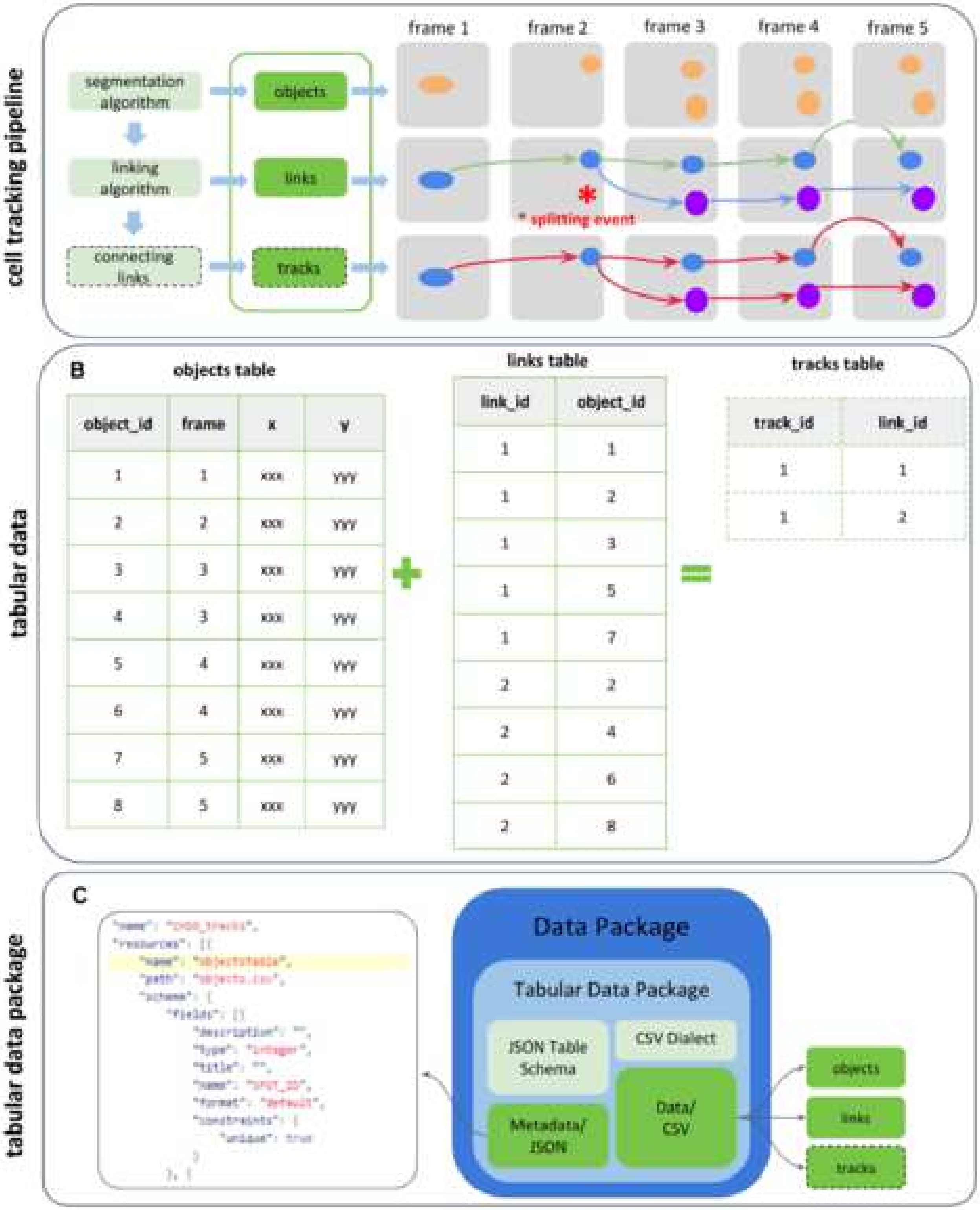

Figure 5

Click here to access/download;Figure;Figure_05_biotracks_API.png

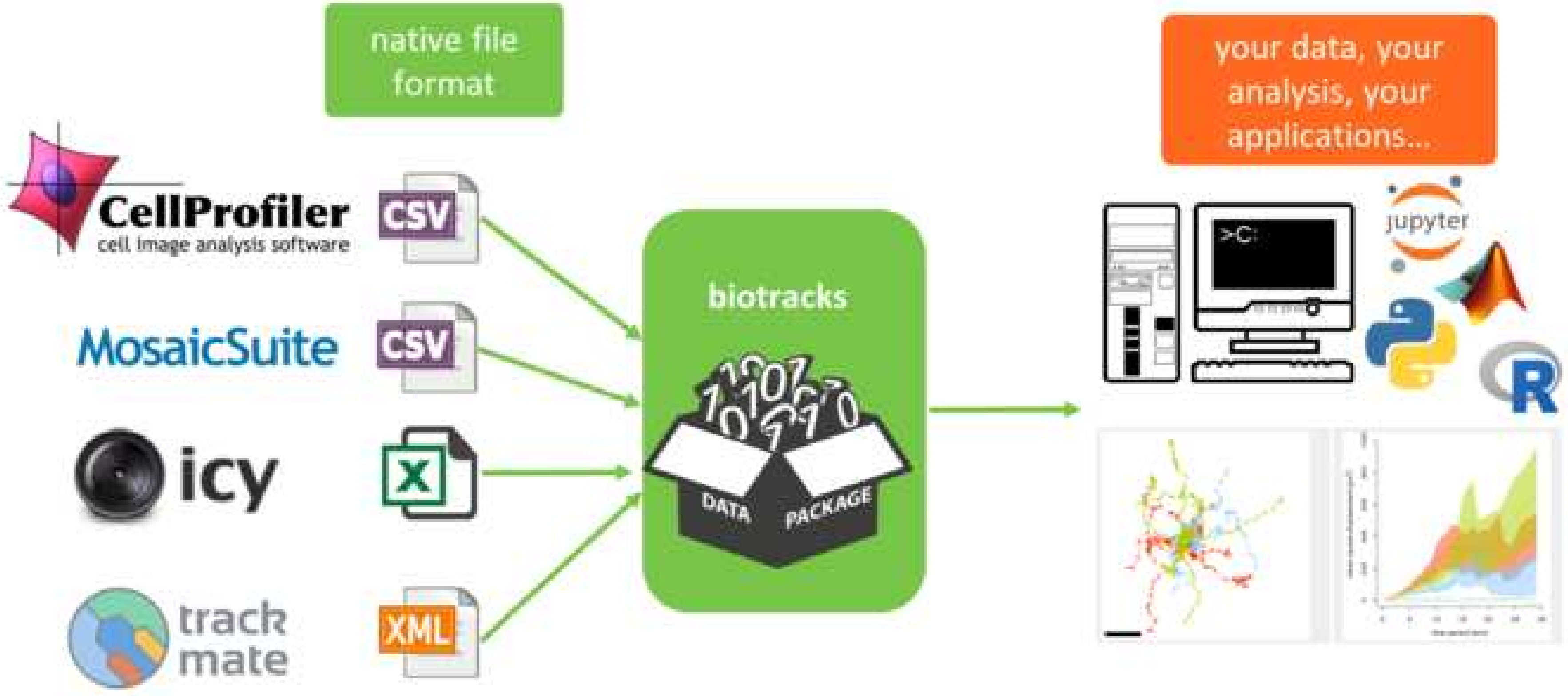

Response to Reviewers Click here to access/download;Personal Cover;CMSO manuscript response to reviewers.docx

Dear Editor,

Thank you very much for the review reports.

We have addressed each of the comments below, and indicated the changes performed to the manuscript. We hope that in view of these changes, the manuscript is ready for acceptance. Reviewers comments appear in blue, our replies are interleaved in black.

Reviewer reports:
Reviewer #1: The authors present their work on creating community-driven standards for reporting and sharing data from cell migration experiments under the FAIR principles. They present specifications for cell migration data at three different levels: the minimal requirements for reporting experiment and imaging conditions using controlled vocabularies (and ontologies) and a data format. The authors provide concrete software implementations for all three levels. They further demonstrate an application of their standards and discuss typical use cases.

This work addresses an essential issue by proposing much-needed standards for reporting and exchanging data from cell migration experiment. I have worked with cell migration data for some years now and thus, very much welcome this valuable contribution from the authors (and the Cell Migration Standardisation Organization) to the community. This contribution is a crucial step towards pushing the field of cell migration analysis forward by enabling scientists to manage, exchange, reuse and mine cell tracking data. The paper is very well written and presents the ideas and concepts clearly and in enough detail. I strongly recommend accepting this manuscript.

We thank Reviewer #1 for the positive comments about our work.

Reviewer #2: Does the manuscript adhere to the journal's guidelines on minimum standards of reporting?
Yes

1) Are the methods appropriate to the aims of the study, are they well described, and are necessary controls included?

The aim of the paper was to establish an adaptable and open data ecosystem for cell migration research community. MIACME framework presented here addressed that aim by implementing minimal information for experiments, controlled vocabularies and data formats.

Incorporation of existing software like ISA, OME, CellProfiler etc. to the ecosystem through API not only increased the acceptance of MIACME but also added value to aforementioned software. All three parts of the framework is well described and all associated resources are well documented and available through Github. This open environment approach will definitely benefit researchers working on cell migration and beyond. This will not only increase the quality and quantity of image derived cellular data but also will fulfil FAIR principles.
However from the manuscript it was not clear how standardised documentation of experimental variables through MIACME framework could increase reproducibility?

We agree with the reviewer that the manuscript has not focused on showing how the MIACME framework could increase reproducibility, but we rely on previous studies showing how reporting guidelines have helped in this area, as detailed below.

The aim of the CMSO is to standardise the *reporting terms and process* of the experimental metadata variables, without limiting labs in their choice of experimental setups. MIACME is a *reporting guideline* that guides the researcher in describing their cell migration experiments, encourages the use of standardised terminology and supports the creation of structured metadata about the data being produced in the experiment.

There have been several studies investigating the efficacy of reporting guidelines to improve reporting and reproducibility. For example, these studies show that the use of checklists in manuscripts can improve the publication quality:

- Turner, L., Shamseer, L., Altman, D.G. *et al.* Does use of the CONSORT Statement impact the completeness of reporting of randomised controlled trials published in medical journals? A Cochrane review[a] . *Syst Rev* **1,** 60 (2012). https://doi.org/10.1186/2046-4053-1-60
- Thomas Fuller, Jaime Peters, Mark Pearson, and Rob Anderson, 2014: Impact of the Transparent Reporting of Evaluations With Nonrandomized Designs Reporting Guideline: Ten Years On American Journal of Public Health **104**, e110_e117, https://doi.org/10.2105/AJPH.2014.302195

A survey by Nature in 2016 (Baker, M. 1,500 scientists lift the lid on reproducibility. *Nature* **533,** 452–454 (2016).) showed that 69% of researchers interviewed considered that journals enforcing standards, such as reporting guidelines, improves reproducibility.

Journals such as PLoS ONE have adopted checklists and analysed the state of adoption by other publishers (https://blogs.plos.org/everyone/2017/06/14/promoting-reproducibility/).

To address these comments, we expanded the text to point to previous studies showing how reporting guidelines improve experimental reporting and reproducibility, adding the following sentence and citations to the section “Minimal reporting guidelines and controlled vocabularies”:

"Reporting guidelines, when enforced by journals, are an important factor to boost reproducibility according to 69% of researchers surveyed by Nature[15] and have also been proven to improve the quality of experimental reporting[16]."

Where the two new references are:

15. *Baker, M. 1,500 scientists lift the lid on reproducibility. Nature **533,** 452–454 (2016).*

16. *Turner, L., Shamseer, L., Altman, D. G., Schulz, K. F. & Moher, D. Does use of the CONSORT Statement impact the completeness of reporting of randomised controlled trials published in medical journals? A Cochrane review. Syst. Rev. **1,** 60 (2012).*

We have also added some clarifying text in the discussion section (see the revised manuscript with track changes).

Particularly how the degree of experimental variations can be identified and measured across different laboratories.

Researchers within the MULTIMOT project are currently performing an experimental study to come up with solutions to these questions as well as a characterization of the major technical sources of variation. However, this study is not yet complete and we cannot provide a full answer for this at this moment.

Lastly on the data format, it was not clear how different levels of object identity (whole cell to subcellular components) can be linked together in different spatiotemporal resolution.

As regards the data format, logically every subcellular component would have coordinates associated with their detection. If the microscope is of sufficiently high-resolution such that the algorithm can separate and segment these components, it would subsequently also be able to link these coordinates together across the time frames into subcellular component tracks. That could raise the question of which coordinates are cells and which are components, when looking at the data table. In that case, it will make sense to add a column to the biotracks 'objects' table with the component name. This concept already exists in for example the tracking software CellProfiler, which distinguishes primary, secondary and tertiary objects (http://cellprofiler-manual.s3.amazonaws.com/CellProfiler-3.0.0/modules/objectprocessing.html#identifyprimaryobjects).

2) Are the conclusions adequately supported by the data shown?

Open cell migration data format and associated tools presented here support the principles of FAIR at one end and the other brings together a wide range of image analysis tools (e.g. CellProfiler) and data formats (BioFormat) together. The case study presented with Masuzzo et al. work provided evidence of applicability of the MIACME framework. Moreover the consortium captured users feedback through series of workshops and online discussion, which supports the arguments presented in relation to users adaptation and sustainability.

3) Please indicate the quality of language in the manuscript. Does it require a heavy editing for language and clarity?

Quality of language was good, ***however Figure 1 and 2 needs editing. For Figure 1, text in bottom box and left hand size box were cut-off. Not sure whether it is due to pdf conversion. For Figure 2 some of the connecting lines are not in alignment. Also the image quality is poor in contrast to figure in Github.***

When checking Figure 1, we could not see anything out of order and are not sure if what the reviewer saw may have been due to the PDF conversion. In any case, we are happy to provide a high quality version of the figure to the journal.

We have updated the design of Figure 2 according to the reviewer's comment.

4) Are you able to assess all statistics in the manuscript, including the appropriateness of statistical tests used?
Yes.

We thank again all the comments by reviewers, which have helped us improve the manuscript, and hope it is now deemed ready for publication.

Kind regards,

Alejandra Gonzalez-Beltran and Gwendolien Sergeant on behalf of the authors
