# Supplementary material for: Community standards for open cell migration data
Source: Gigascience. 2020 May 12;9(5):giaa041. doi: 10.1093/gigascience/giaa041 (PMC7317087; doi:10.1093/gigascience/giaa041)
Supplement: giaa041_Supplemental_File [file giaa041_supplemental_file.pdf]

Supplementary Table 1: A MIACME-annotated cell migration study.

The cell migration study from Masuzzo *et al*.[30] described and annotated using the cell-migration-specific part of the MIACME guidelines (version 1.1). Highlighted in gray, we show elements that are part of the MIACME requirements but were not reported in the original paper.

**Experimental setup**	
**Entity**	**Term [CV]**
Basic Approach	*In vitro* design [OBI:0001285]
Cell Model	Cell type:<br>cell line cell [CLO:0000001]
	Specific Cell Details:<br>B-cell [CL:0000236]<br>Ba/F3 cell [CLO:0001842]
	Organism: Mus musculus [NCBI:txid10090]
Assay	Assay type: Single-cell migration [*]<br>Cell Input: single cells [*]
Treatment	Factor: Bcr-Abl fusion protein [PR:000044437] oncogene variants[58]
	Factor: ROCK inhibitor y-27632 [CHEBI:75393]<br>Factor level: 10 µM
Microenvironment	BD Matrigel™ Basement Membrane [GO:0005604,NCIT_C19834] Matrix

Medium	RPMI 1640 + FBS [fetal bovine serum]<br>[CCONT:0000048][+]
**Imaging condition**	
**Entity**	**Term [CV]**
Imaging Modality	Phase-contrast [ERO:0001636]
Image Sequence Type	Time series / Time-lapse [FBbi_00000249]
Observation Period	6
Observation Period Unit	hours
Time Series Interval	1.5
Time Series Interval Unit	minutes
Objective Lens	Manufacturer: Olympus<br>Acronym: LCPLFLN<br>Magnification: 20x
Numerical Aperture	0.45

Pixel	Pixel dimension order: XYTZC<br><br>Pixel type: int16<br><br>Pixel size X: 1376<br><br>Pixel size Y: 1038<br><br>Pixel size T: 240<br><br>Pixel size C: 1<br><br>physicalSizeX: 0.645<br><br>physicalSizeXUnit: µm<br><br>physicalSizeY:0.645<br><br>physicalSizeYUnit: µm
Channel Definitions	Channel #1: Cells [CL:0000000]
**Data**	
**Entity**	**Term [CV]**
rawImagesDataset<br><br>description<br><br>numberOfFiles	144 raw images x 12 wells<br><br>1728
processedImagesDataset<br><br>description<br><br>keyword	Segmented images/images with tracks<br><br>[NCIT_C80146]
extractedFeaturesDataset<br><br>description<br><br><br>keyword<br><br>keyword<br><br>keyword	Extracted features in the study.<br><br>X coordinate [NCIT_C44477]<br><br>Y coordinate [NCIT_C44478]<br><br>Timepoint [NCIT_C68568]

[*] a CV is not present in literature to describe the term and will be addressed by CMSO.

[+] the cell culture ontology has the term we need, but it has been down for some time; the developer indicated that due to GDPR issues, they are revising the webpages and might be down for a while. If not restored, this term would be a candidate for creation within a CMSO ontology.
