# Supplementary material for: Community standards for open cell migration data
Source: Gigascience. 2020 May 12;9(5):giaa041. doi: 10.1093/gigascience/giaa041 (PMC7317087; doi:10.1093/gigascience/giaa041)
Supplement: giaa041_Response_to_Reviewer_Comments_Original_Submission [file giaa041_response_to_reviewer_comments_original_submission.pdf]

Author's Response To Reviewer Comments

Close

We have responded to each of the reviewer comments in the Response Document added to the submission, and indicated the changes performed to the manuscript. We thank the reviewers again for their comments, which have helped us improve the manuscript.

Close
