# Supplementary material for: Community standards for open cell migration data
Source: Gigascience. 2020 May 12;9(5):giaa041. doi: 10.1093/gigascience/giaa041 (PMC7317087; doi:10.1093/gigascience/giaa041)
Supplement: giaa041_Reviewer_1_Report_Original_Submission — Nico Scher -- 12/5/2019 Reviewed [file giaa041_reviewer_1_report_original_submission.pdf]

Reviewer Report

Title: Community Standards for Open Cell Migration Data

Version: Original Submission **Date:** 12/5/2019

Reviewer name: Nico Scher

Reviewer Comments to Author:

The authors present their work on creating community-driven standards for reporting and sharing data from cell migration experiments under the FAIR principles. They present specifications for cell migration data at three different levels: the minimal requirements for reporting experiment and imaging conditions using controlled vocabularies (and ontologies) and a data format. The authors provide concrete software implementations for all three levels. They further demonstrate an application of their standards and discuss typical use cases.

This work addresses an essential issue by proposing much-needed standards for reporting and exchanging data from cell migration experiment. I have worked with cell migration data for some years now and thus, very much welcome this valuable contribution from the authors (and the Cell Migration Standardisation Organization) to the community. This contribution is a crucial step towards pushing the field of cell migration analysis forward by enabling scientists to manage, exchange, reuse and mine cell tracking data. The paper is very well written and presents the ideas and concepts clearly and in enough detail. I strongly recommend accepting this manuscript.

Methods

Are the methods appropriate to the aims of the study, are they well described, and are necessary controls included? Choose an item.

Conclusions

Are the conclusions adequately supported by the data shown? Choose an item.

Reporting Standards

Does the manuscript adhere to the journal's guidelines on [minimum standards of reporting?](#) Choose an item.

Choose an item.

Statistics

Are you able to assess all statistics in the manuscript, including the appropriateness of statistical tests used? Choose an item.

Quality of Written English

Please indicate the quality of language in the manuscript: Choose an item.

Declaration of Competing Interests

Please complete a declaration of competing interests, considering the following questions:

- Have you in the past five years received reimbursements, fees, funding, or salary from an organisation that may in any way gain or lose financially from the publication of this manuscript, either now or in the future?
- Do you hold any stocks or shares in an organisation that may in any way gain or lose financially from the publication of this manuscript, either now or in the future?
- Do you hold or are you currently applying for any patents relating to the content of the manuscript?
- Have you received reimbursements, fees, funding, or salary from an organization that holds or has applied for patents relating to the content of the manuscript?
- Do you have any other financial competing interests?
- Do you have any non-financial competing interests in relation to this paper?

If you can answer no to all of the above, write 'I declare that I have no competing interests' below. If your reply is yes to any, please give details below.

I declare that I have no competing interests

I agree to the open peer review policy of the journal. I understand that my name will be included on my report to the authors and, if the manuscript is accepted for publication, my named report including any attachments I upload will be posted on the website along with the authors' responses. I agree for my report to be made available under an Open Access Creative Commons CC-BY license (http://creativecommons.org/licenses/by/4.0/). I understand that any comments which I do not wish to be included in my named report can be included as confidential comments to the editors, which will not be published.

Choose an item.

To further support our reviewers, we have joined with Publons, where you can gain additional credit to further highlight your hard work (see: https://publons.com/journal/530/gigascience). On publication of this paper, your review will be automatically added to Publons, you can then choose whether or not to claim your Publons credit. I understand this statement.

Yes Choose an item.
