# Supplementary material for: Community standards for open cell migration data
Source: Gigascience. 2020 May 12;9(5):giaa041. doi: 10.1093/gigascience/giaa041 (PMC7317087; doi:10.1093/gigascience/giaa041)
Supplement: giaa041_Reviewer_2_Report_Original_Submission — Imtiaz Khan -- 12/16/2019 Reviewed [file giaa041_reviewer_2_report_original_submission.pdf]

Reviewer Report

Title: Community Standards for Open Cell Migration Data

Version: Original Submission Date: 12/16/2019

Reviewer name: Imtiaz Khan

Reviewer Comments to Author:

Does the manuscript adhere to the journal's guidelines on minimum standards of reporting?
Yes
1) Are the methods appropriate to the aims of the study, are they well described, and are necessary controls included?
The aim of the paper was to establish an adaptable and open data ecosystem for cell migration research community. MIACME framework presented here addressed that aim by implementing minimal information for experiments, controlled vocabularies and data formats. Incorporation of existing software like ISA, OME, CellProfiler etc. to the ecosystem through API not only increased the acceptance of MIACME but also added value to aforementioned software. All three parts of the framework is well described and all associated resources are well documented and available through Github. This open environment approach will definitely benefit researchers working on cell migration and beyond. This will not only increase the quality and quantity of image derived cellular data but also will fulfil FAIR principles. However from the manuscript it was not clear how standardised documentation of experimental variables through MIACME framework could increase reproducibility? Particularly how the degree of experimental variations can be identified and measured across different laboratories. Lastly on the data format, it was not clear how different levels of object identity (whole cell to subcellular components) can be linked together in different spatiotemporal resolution.
2) Are the conclusions adequately supported by the data shown?
Open cell migration data format and associated tools presented here support the principles of FAIR at one end and the other brings together a wide range of image analysis tools (e.g. CellProfiler) and data formats (BioFormat) together. The case study presented with Masuzzo et al. work provided evidence of applicability of the MIACME framework. Moreover the consortium captured users feedback through series of workshops and online discussion, which supports the arguments presented in relation to users adaptation and sustainability.
3) Please indicate the quality of language in the manuscript. Does it require a heavy editing for language and clarity?
Quality of language was good, however Figure 1 and 2 needs editing. For Figure 1, text in bottom box and left hand size box were cut-off. Not sure whether it is due to pdf conversion. For Figure 2 some of the connecting lines are not in alignment. Also the image quality is poor in contrast to figure in Github.
4) Are you able to assess all statistics in the manuscript, including the appropriateness of statistical tests used?
Yes.

Methods

Are the methods appropriate to the aims of the study, are they well described, and are necessary controls included? Choose an item.

Conclusions

Are the conclusions adequately supported by the data shown? Choose an item.

Reporting Standards

Does the manuscript adhere to the journal's guidelines on minimum standards of reporting? Choose an item.

Choose an item.

Statistics

Are you able to assess all statistics in the manuscript, including the appropriateness of statistical tests used? Choose an item.

Quality of Written English

Please indicate the quality of language in the manuscript: Choose an item.

Declaration of Competing Interests

Please complete a declaration of competing interests, considering the following questions:

- Have you in the past five years received reimbursements, fees, funding, or salary from an organisation that may in any way gain or lose financially from the publication of this manuscript, either now or in the future?
- Do you hold any stocks or shares in an organisation that may in any way gain or lose financially from the publication of this manuscript, either now or in the future?
- Do you hold or are you currently applying for any patents relating to the content of the manuscript?
- Have you received reimbursements, fees, funding, or salary from an organization that holds or has applied for patents relating to the content of the manuscript?
- Do you have any other financial competing interests?
- Do you have any non-financial competing interests in relation to this paper?

If you can answer no to all of the above, write 'I declare that I have no competing interests' below. If your reply is yes to any, please give details below.

'I declare that I have no competing interests

I agree to the open peer review policy of the journal. I understand that my name will be included on my report to the authors and, if the manuscript is accepted for publication, my named report including any attachments I upload will be posted on the website along with the authors' responses. I agree for my report to be made available under an Open Access Creative Commons CC-BY license (http://creativecommons.org/licenses/by/4.0/). I understand that any comments which I do not wish to be included in my named report can be included as confidential comments to the editors, which will not be published.

Choose an item.

To further support our reviewers, we have joined with Publons, where you can gain additional credit to further highlight your hard work (see: https://publons.com/journal/530/gigascience). On publication of this paper, your review will be automatically added to Publons, you can then choose whether or not to claim your Publons credit. I understand this statement.

Yes Choose an item.
